# MicroRNA miR-330-3p suppresses the progression of ovarian cancer by targeting RIPK4

**DOI:** 10.1080/21655979.2021.1871817

**Published:** 2021-01-25

**Authors:** Li Cai, Lu Ye, Xiaoqing Hu, Wenfeng He, Debao Zhuang, Qi Guo, Kuanyong Shu, Youkun Jie

**Affiliations:** a Department of Oncology, Jiangxi Maternal and Child Health Hospital, Nanchang, Jiangxi, China; b Department of Pathology, Jiangxi Maternal and Child Health Hospital, Nanchang, Jiangxi, China; c Jiangxi Key Laboratory of Molecular Medicine, The Second Affiliated Hospital of Nanchang University, Nanchang, Jiangxi, China; Department of Psychiatry, Jiangxi Mental Hospital/Affiliated Mental Hospital of Nanchang University, Nanchang, Jiangxi, China

**Keywords:** Mir-330-3p, RIPK4, ovarian cancer

## Abstract

Previous studies reported that miR-330-3p was involved in the progression of several cancers, but the potential roles of miR-330-3p in ovarian cancer (OC) were unclear. In the current study, we aimed to explore the expression pattern and functions of miR-330-3p in OC. The expression level of miR-330-3p in OC tissues and cell lines was detected using RT-qPCR. The proliferation, migration and invasion of OC cells were detected using CCK-8 assay and transwell assay, respectively. Bioinformatics analysis and luciferase reporter assay were used to analyze the targeted binding site of miR-330-3p and RIPK4. The results showed that miR-330-3p was significantly downregulated in OC tissues and cell lines. Overexpression of miR-330-3p inhibited the proliferation, migration and invasion of OC cells. Mechanistically, a dual-luciferase reported assay showed that RIPK4 is a target gene of miR-330-3p. Furthermore, rescue experiments revealed that miR-330-3p suppressed the proliferation, migration and invasion of OC cells by targeting RIPK4. In summary, our findings indicated that miR-330-3p suppressed the progression of OC by targeting RIPK4. Our results indicated that miR-330-3p/RIPK4 axis might act as a novel therapeutic target for OC treatment.

## Graphical abstract

**Figure:**
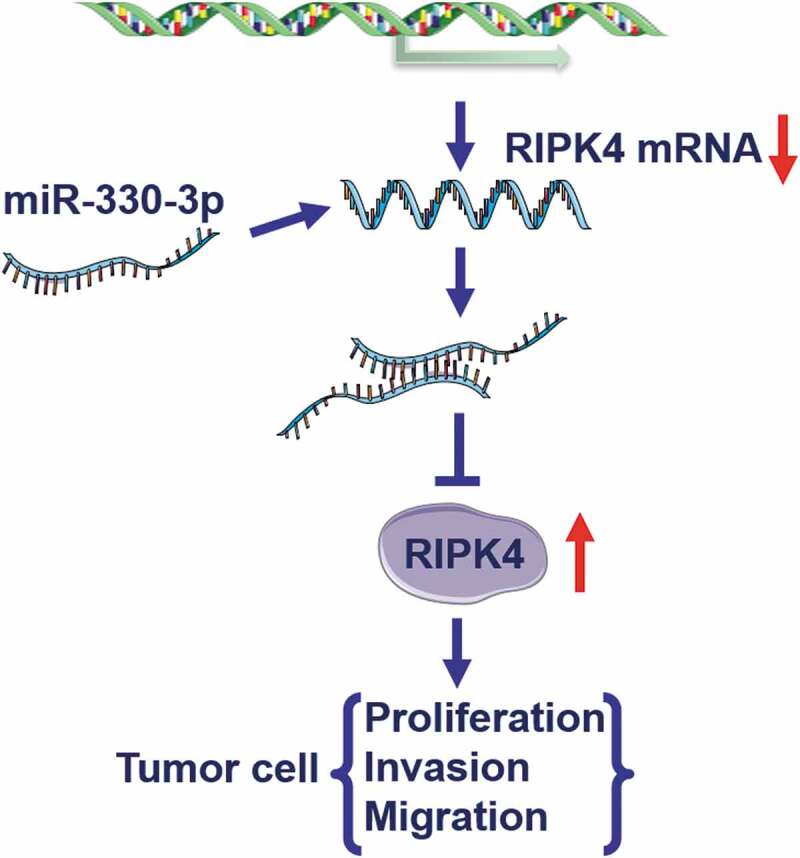

## Introduction

Ovarian cancer (OC) is one of the most common causes of cancer-related death in women [1,2]. As the ‘silent killer’, over 70% of OC can not be diagnosed until it has developed into an advanced stage [3]. Over the past 20 years, the incidence of OC has fallen by less than 1%, but the mortality and 5-year survival of OC have remained largely unchanged [4]. Regardless of great advancements into exploring the mechanism of OC, its pathogenesis is largely unknown. Generally, operation together with chemotherapy was the main method for OC treatment [5]. However, these treatments usually hardly prevent the recurrence and metastasis of OC, and the 5-year survival rate for patients with OC is still less than 30% [5,6]. Therefore, it is essential to clarify the pathogenesis of OC, which contributes to providing novel therapeutic targets for OC treatment.

Accumulating evidence indicated that microRNAs (miRNAs) participated in regulating the proliferation, apoptosis, invasion, migration and phenotypic transformation of cancer cells by binding to 3ʹ-untranslated region (3ʹ-UTRs) of the target mRNA [7,8]. Also, previous studies found that miR-330-3p played important roles in the progression of various cancers. For instance, inhibition of miR-330-3p promoted the progression of non-small-cell lung cancer via RASSF1. Moreover, upregulation of miR-330-3p restricted the proliferation, migration and invasion of laryngeal squamous cell carcinoma cells through targeting Tra2β to inhibit Akt activation [9]. Guan et al. found that miR-330-3p could act as a tumor suppressor in gastric cancer by targeting MSI1 [10]. Similar results were identified in colorectal cancer [11] and liver cancer [12]. Regardless of the important functions of miR-330-3p in onset and development of other cancers, the potential roles of miR-330-3p in OC were largely unknown.

RIPK4, as a member of receptor-interacting kinase proteins (RIPKs) family, was first described as an unidentified protein interacting with PKC-β1 Pectin and PKC δ [13,14]. As a necessary receptor for intracellular and extracellular stress signal transduction, RIPK4 plays an important role in inflammatory immune response injury and induced cell activation and death [15]. Previous studies also indicated that RIPK4 acted as an oncogene in several cancers. Gong et al. found that RIPK4 promoted the progression of nasopharyngeal carcinoma through activating NF-kB signaling [16]. Meanwhile, Qi and coworkers revealed that RIPK4 facilitated the migration and invasion of pancreatic cancer cells via the RAF1/MEK/ERK pathway [17]. Susan and colleagues demonstrated that RIPK4 contributed to lymph node metastasis and predicted favorable prognosis in cervical cancer [18]. Until now, there were no studies exploring the roles of RIPK4 in OC.

In the current study, we try to explore the potential roles and possible mechanisms of miR-330-3p in OC. We found that miR-330-3p was significantly downregulated in OC tissues and cells. Moreover, overexpression of miR-330-3p could inhibit the proliferation, migration and invasion of OC cells. Bioinformatics analysis and luciferase reported assay showed that RIPK4 was a target gene of miR-330-3p. Furthermore, rescue experiments revealed that miR-330-3p suppressed the proliferation, migration and invasion of OC cells by targeting RIPK4. To sum up, our findings indicated that miR-330-3p suppressed the progression of OC by targeting RIPK4. Our results indicated that miR-330-3p/RIPK4 axis participated in the progression of OC, which might act as a novel therapeutic target for OC treatment.

## Materials and methods

### Patients and tissues samples

A total of 30 paired OC tissue specimens and adjacent normal tissue specimens were collected from OC patients at Jiangxi Maternal and Child Health Hospital (Jiangxi, China). All tumor specimens were diagnosed by pathological examination. Clinicopathological characteristics of patients are recorded in Table 1. Samples were immediately frozen in liquid nitrogen. All patients had not received chemotherapy or radiation therapy prior to surgery. Besides, all patients had signed consent form before this research, which was approved by Ethics Committee of our hospital.

**Table 1.** Association of miR-330-3p expression with clinicopathological factors of the ovarian cancer patients

Clinicopathological variables		*n*	miR-330-3p expression		*P* value
			Low (*n* = 19)	High (*n* = 11)	
Age, years	≤50	12	7	5	0.712
	50	18	12	6	
Histological type	Serous	21	13	9	0.672
	Non‑serous	8	6	2	
Pathological grade	1–2	8	4	4	0.417
	3	22	15	7	
Clinical stage	I and II	13	5	8	0.023*
	III and IV	17	14	3	

### Cell culture and cell transfection

Human normal ovarian surface epithelial cells (IOSE80) and three OC cell lines including OVCAR3, SKOV3 and ES-2 were purchased from the Bank of Type Culture Collection of Chinese Academy of Sciences (Shanghai, China). Cells were cultured in Dulbecco modified Eagle medium (DEME) (Invitrogen Carlsbad, CA) supplemented with 10% fetal bovine serum (FBS) in a humidified atmosphere of 5% CO2 incubator at 37°C. Cells were cultured in 6-well plates for at least 24 h before transfection. miR-330-3p mimic, mimic NC, oe-RIPK4 and NC were purchased from GenePharma (Shanghai, China) and transfected into OC cell lines by Lipofectamine 2000 (Thermo Fisher Scientific, Inc.) following the manufacturer`s instructions.

### Real-time quantitative reverse transcription-polymerase chain reaction (RT-qPCR)

Total RNA from OC tissues and cells was extracted using TRIzol reagent (Invitrogen, Carlsbad, CA, USA) with a RecoverAll™ Total Nucleic Acid Isolation kit (Ambion, Foster City, CA, USA), and reverse transcription reactions were performed using the Prime Script™ RT reagent kit following the manufacturer’s instructions (Takara, Dalian, China). U6 snRNA was used as internal references for the detection of miR-330-3p. Subsequently, RT-qPCR analyses for RIPK4 and the normalization control gene GAPDH were performed with SYBR Premix Ex Taq (Takara Bio, Shiga, Japan). Relative expression levels of miR-330-3p and RIPK4 were calculated based on the comparative CT method. The primer sequences are as follows:

RIPK4 (Forward:GATCTCCGGTTCCGAATCATC;

Reverse: TCAGAAATCTTGACGTGGTAGTG)

GAPDH (Forward: GGAGCGAGATCCCTCCAAAAT;

Reverse: GGCTGTTGTCATACTTCTCATGG)

miR-330-3p(Forward: GCGGCGGGCAAAGCACACGGCC;

Reverse: ATCCAGTGCAGGGTCCGAGG)

U6 (Forward: GCTTCGGCAGCACATATACTAAAAT;

Reverse: CGCTTCACGAATTTGCGTGTCAT).

### Cell proliferation assay

After being detached from culture dishes by trypsinization, cells were transfected with miR-330-3p mimics or NC and seeded in 96-well plates (2 × 10^3^ cells per well). Cell proliferation was assessed by MTT kit (Sigma, St Louis, MO, USA). Optical density value (OD value) at the wavelength of 490 nm was measured using a Microplate Reader (Bio-Rad, Hercules, CA, USA) after 4 hours later of incubation with 20 μL MTT (Thermo Fisher Scientific, Waltham, MA, USA).

### Cell migration and invasion assay

Transwell chambers were used to assess the migration and invasion potentials of OC cells. Cancer cell migration assay was performed with transwell chambers without Matrigel (Millipore, Billerica, MA, USA), and the transwell inserts were coated with Matrigel (BD Biosciences, Franklin Lakes, NJ, USA) were used in cell invasion assay. Briefly, transfected OC cells in the logarithmic growth phase (10^4–^10^5^ cells per well) were seeded to the upper chambers of Transwell plates suspended in 200 μL serum-free DMEM medium. Then, 500 μL DMEM medium containing 10% FBS was added to the lower chamber and incubated at 37°C for 24 h. Non-migrated or non-invading cells remaining in the upper chamber were scrubbed with cotton swabs. Then, membranes were fixed with 4% paraformaldehyde and stained with 1% crystal violet for 30 min. Five random areas were counted per chamber under an inverted microscope (Olympus, Tokyo, Japan).

### Luciferase reporter assay

The target miRNA of RIPK4 were analyzed using bioinformatics online software (Tarbase, Starbase, and TargetScan) and the result indicated RIPK4 was a putative target for miR-330-3p. Luciferase reporter assays were carried out to test the interaction between the RIPK4 and miR-330-3p using the Dual-Luciferase Reporter Assay System (Promega, Madison, WI, USA). The RIPK4 mutant-type (RIPK4-mut) and RIPK4 wild-type (RIPK4-wt) vectors were synthetized by RIPK4 cDNA fragment insertion which contained mutated or wild at binding sites of miR-330-3p into the pGL3 luciferase reporter vectors using Lipofectamine 2000 (Invitrogen). The cells were co-transfected with pGL3 reporter luciferase vector, which contains the 3ʹ-UTR sequence of RIPK4-mut and RIPK4-wt and miR-330-3p mimics or negative control using Lipofectamine 2000 (Invitrogen). After 48 h following transfection, cell lysated were collected and the relative luciferase activity was assessed with the Dual-Luciferase Reporter Assay System (Promega, Madison WI, USA) following the instructions of manufacture.

### Western blot assay

Total proteins were loaded into SDS-PAGE for separation after extraction and concentration measurement and were transferred onto a PVDF membrane. For detecting target proteins, the membrane was blocked with 5% nonfat milk powder at room temperature for 1 h and then probed with corresponding primary antibodies. Primary antibodies against RIPK4 (ab203541, Abcam, Cambridge, UK) and GAPDH (ab8245, Abcam, Cambridge, UK) were utilized in this study. Finally, the membrane was incubated with appropriate secondary antibody (Abcam). The expression of proteins was visualized using enhanced chemiluminescence (ECL).

### Statistical analysis

Data were presented as the mean ± standard deviation (SD) calculated from at least three independent experiments. Statistical analysis was performed using SPSS 22.0 software (SPSS, Chicago, IL, USA). Student’s t-test or two-way analysis of variance (ANOVA) were used to explore the statistically significant differences. The correlation between miR-330-3p and RIPK4 mRNA expression in OC tissues was estimated using Spearman’s correlation analysis. *P* value <0.05 was considered as statistically significant.

## Results

### miR-330-3p was downregulated in OC tissue specimens and cell lines

**Figure 1.**
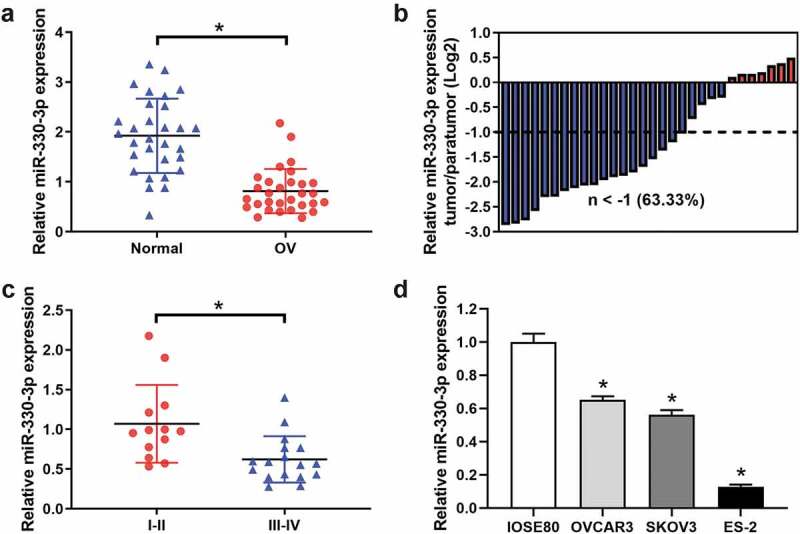
The expression of miR-330-3p was downregulated in OC samples and cell lines. (a) The expression level of miR-330-3p in 30 paired OC tissues and adjacent non-tumor tissues, *P < 0.05. (b) The relative miR-330-3p expression was assessed in OC tissues. (c)The expression level of miR-330-3p in different clinical stage in OC tissues (III–IV vs. I–II), *P < 0.05. (d) Expression levels of miR-330-3p in OC cell lines, *P < 0.05

The expression levels of miR-330-3p in OC tissues and cell lines were examined by RT-qPCR. The results indicated that the expression level of miR-330-3p was significantly downregulated in OC tissues as compared with those in adjacent non-tumor tissues (Figure 1a). The miR-330-3p expression levels were lower in 63.33% of OC tumor tissue samples (Figure 1b). Also, we explored the expression level of miR-330-3p in different clinical stages of OC. We found that the expression of miR-330-3p was significantly lower in III–IV OC tissues as compared with I–II (Figure 1c and Table 1). In addition, miR-330-3p was frequently downregulated in the OC cell lines OVCAR3, SKOV3 and ES2 cells as compared to human normal ovarian epithelial cell line (IOSE80) (Figure 1d). Interestingly, the expression of miR-330-3p in ES-2 cells was the lowest among all the OC cell lines, so we chose it in subsequent experiments.

### Overexpression of miR-330-3p suppressed the proliferation, migration and invasion of OC cells

**Figure 2.**
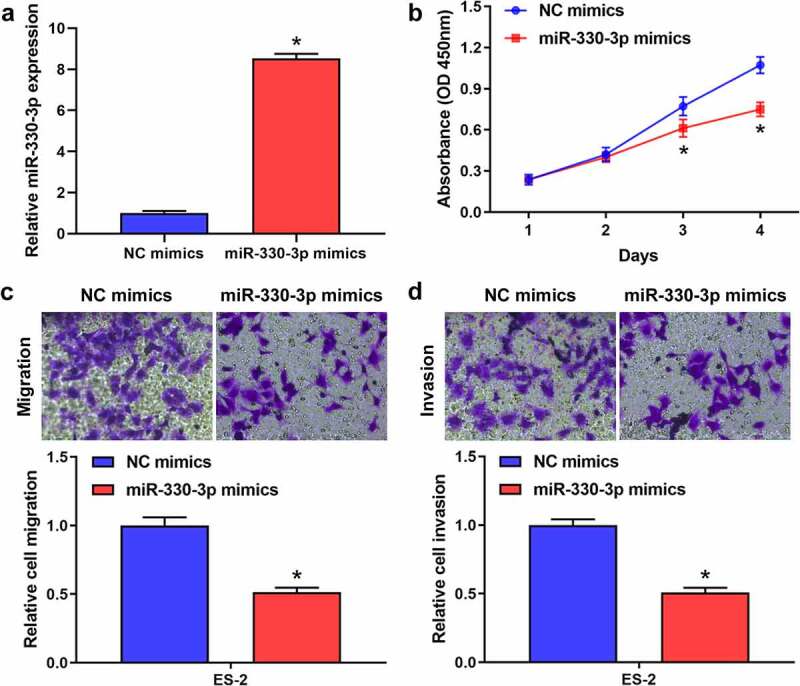
Overexpression of miR-330-3p inhibited ES-2 cell proliferation, migration and invasion. (a) RT-qPCR analysis was performed to assess the expression of miR-330-3p after transfected with miR-330-3p mimics and NC, *P < 0.05. (b) The proliferation of ES-2 cells after transfection was detected by MTT assay, *P < 0.05. (c,d) The migration and invasion of ES-2 cells after transfection was detected by transwell invasion and migration assays, *P < 0.05

To explore the effect of miR-330-3p on the proliferation, migration and invasion of OC cells, ES-2 cells were transfected with miR-330-3p mimics or NC. RT-qPCR showed that miR-330-3p expression was significantly upregulated after transfected with miR-330-3p mimics (Figure 2a). The results of the MTT assay indicated that the proliferation of ES-2 cells was significantly inhibited after overexpressing miR-330-3p (Figure 2b). Transwell assay indicated that overexpression of miR-330-3p dramatically inhibited the migration and invasion of ES-2 cells (showed in Figure 2c, D). Collectively, these findings indicated that miR-330-3p may act as a tumor suppressor in OC.

### RIPK4 acts as a target gene of miR-330-3p

**Figure 3.**
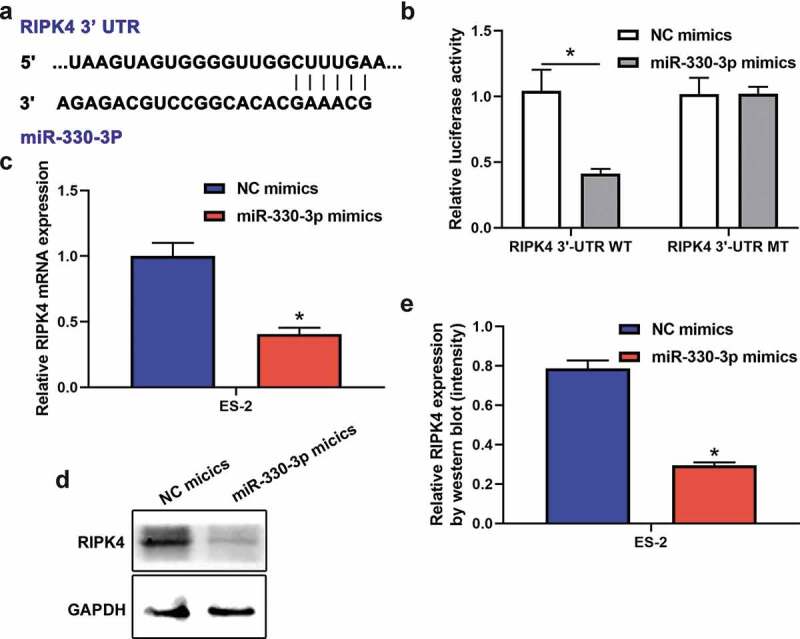
RIPK4 was a direct target of miR-330-3p in ES-2 cells. (a) The binding sites between RIPK4 and miR-330-3p predicted by TargetScan. (b)The binding relationship of RIPK4 to miR-330-3p validated by dual-luciferase reporter gene assay, *P < 0.05. The expression level of RIPK4 was detected using RT-qPCR (c) and western blot (d-e), *P < 0.05

Accumulating evidence indicate that miRNAs exert their functions through binding to target mRNAs. Previous studies identified that RIPK4 acted as a significant oncogene in many cancers [16,19]. Interestingly, RIPK4 was verified to be a potential targeted gene of miR-330-3p [20,21]. Based on these, we try to explore whether RIPK4 was a target gene of miR-330-3p in ovarian cancer. miRNA target analysis using Targetscan identified a complementary region of miR-330-3p in the 3`-UTR of RIPK4 (Figure 3a). Moreover, dual-luciferase reporter assay showed that miR-330p-3p inhibited the luciferase activities in ES-2 cells transfected with RIPK4-WT, but miR-330-3p showed no effect on the mutated RIPK4 3ʹ-UTR fragment (Figure 3b). In addition, RT-qPCR and western blot showed that mRNA and protein expression of RIPK4 were significantly downregulated after transfected with miR-330-3p mimics (Figure 3c–e). Take together, these findings indicated that RIPK4 is a direct target of miR-330-3p.

### Overexpression of miR-330-3p suppressed the proliferation, migration and invasion of OC cells via RIPK4

**Figure 4.**
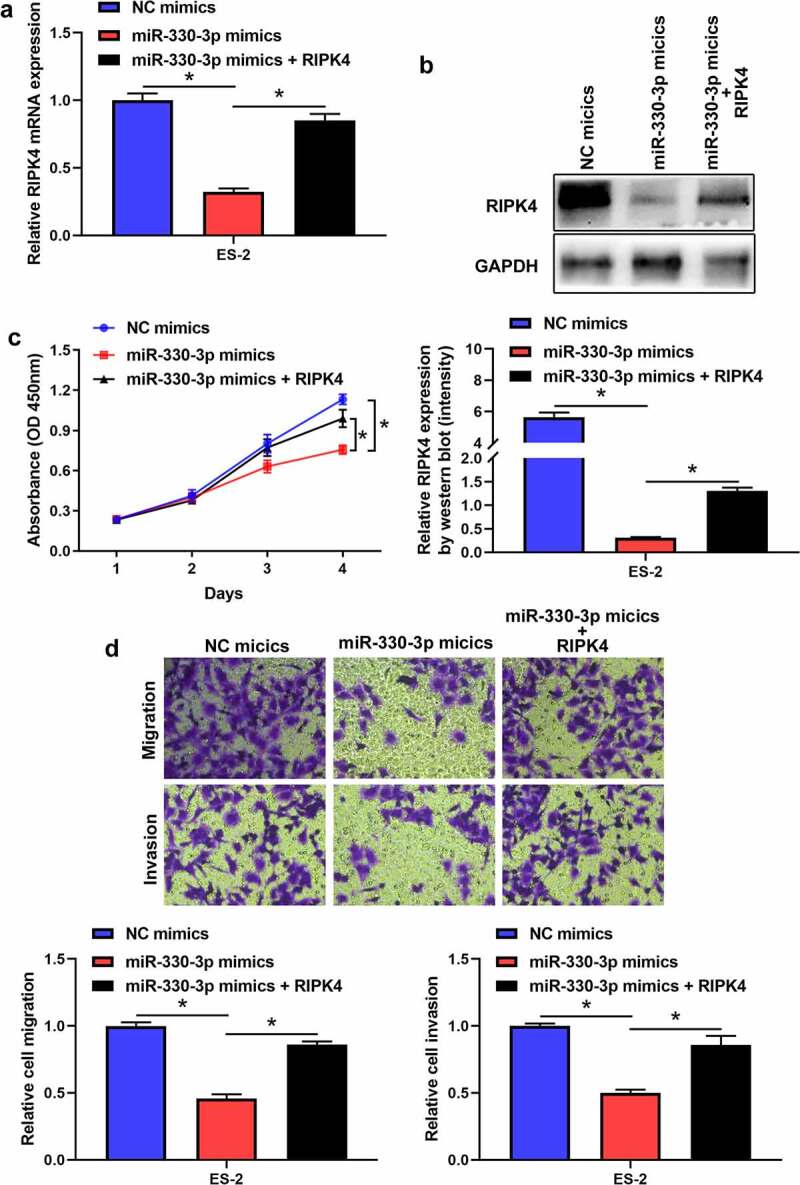
Overexpression of RIPK4 antagonized the effects of miR-330-3p on ES-2 cells. The inhibition effect of miR-330-3p on RIPK4 expression was rescued by RIPK4 overexpression detected using RT-qPCR (a) and western blot (b), *P < 0.05. The inhibition effect of miR-330-3p on cell proliferation, migration and invasion was partly reversed by RIPK4 overexpression detected using MTT (c) and transwell assay(d), *P < 0.05

**Figure 5.**
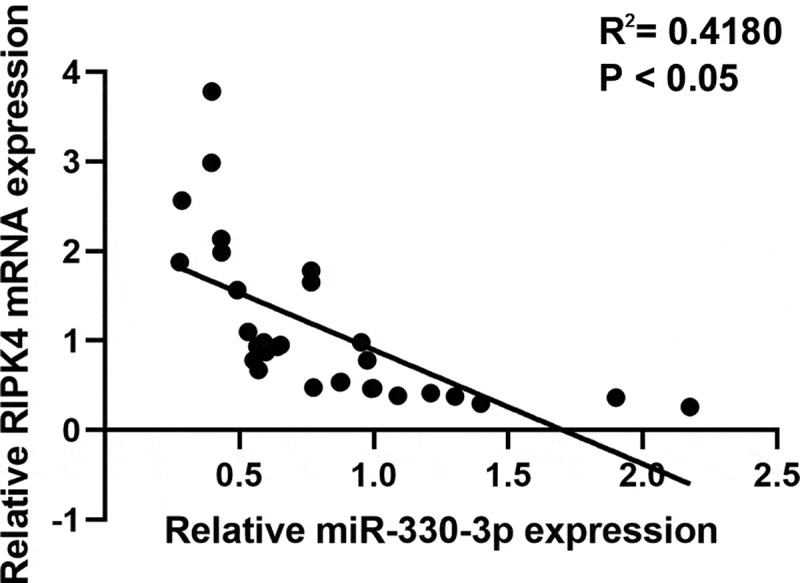
The expression of RIPK4 was inversely correlated with miR-330-3p expression in OV tissues

To further verify whether miR-330-3p induced tumorigenicity via RIPK4, we co-transfected miR-330-3p mimics with the RIPK4 overexpression vector. RT-qPCR and western blot indicated that overexpression of RIPK4 attenuated miR-330-3p-triggered RIPK4 downregulation in ES-2 cells (Figure 4a,b). Furthermore, upregulation of RIPK4 can partly restored miR-330-3p-mediated OC cells’ proliferation, migration and invasion (Figure 4c,d). In addition, the correction analysis showed that high expression of miR-330-3p was associated with low level of RIPK4 (P < 0.05, R^2^ = 0.418) (Figure 5). Taken together, these findings indicated that overexpression of miR-330-3p suppressed the proliferation, migration and invasion of OC cells via RIPK4.

## Discussion

Increasing evidence indicated that miRNAs participated in the development and progression of many cancers including gastric cancer [22], melanoma [23], laryngeal squamous cell carcinoma [9]. However, the potential roles of miR-330-3p in OC were still unknown. In this study, we found that miR-330-3p was significantly downregulated in OC tissues and cells lines. Moreover, miR-330-3p suppressed the proliferation, migration and invasion of OC cells via RIPK4.

Previous studies identified that miR-330-3p acted as a tumor suppressor in many cancers, but the roles of OC were largely unclear. In the current study, we found that miR-330-3p was significantly downregulated in OC tissues and cell lines. Overexpression of miR-330-3p inhibited the proliferation, migration and invasion of OC cells. Mechanistically, a dual-luciferase reported assay showed that RIPK4 is a target gene of miR-330-3p. Furthermore, rescue experiments revealed that miR-330-3p suppressed the proliferation, migration and invasion of OC by targeting RIPK4. RIPK4, located at chromosome band 21q22.3, is a serine/threonine kinase which is a 91.6 kDa protein [24]. As a member of receptor-interacting kinase proteins (RIPKs) family [25], RIPK4 encoded by this gene is a serine/threonine protein kinase that interacts with protein kinase C-delta. But the functions of RIPs members are different due to the difference in the C-terminal domain [26–29]. RIPK4 are widely expressed in various mature tissues and embryos and promotes differentiation and apoptosis through activating nuclear factor-κB (NF-κB), c-Jun N-terminal kinase (c-JNK) signal path [30] and activator protein-1 (AP-1). Recent researches indicated that RIPK4 was associated with the progression of many cancers. Liu *et al.* found that RIPK4 expression was significantly increased in cervical cancer cells and overexpression of RIPK4 was associated with a poor overall survival (OS) and disease-free survival (DFS) [31]. RIPK4 promoted invasion and metastasis of cervical cancer cells by inhibiting expression of 'vimentin, MMP2 and fibronectin [31] which were pivotal molecules of epithelial-mesenchymal transition process [32]. Besides, high-level expression of RIPK4 promoted lymph node metastasis in cervical cancer [18]. Significantly upregulation of RIPK4 was also identified in ovary and skin tumors [33]. Moreover, some studies indicated that overexpression of RIPK4 could lead to resistance of chemotherapy and promote the progression and recurrence of cancers through activating NF-κB and Wnt/β-catenin signal pathways [18]. Interestingly, Huang *et al.* found that RIPK4 knockdown in A2780 and COV434 OC cells inhibited β-catenin accumulation [33]. Actually, β-catenin nuclear accumulation could promote OC progression through activating Wnt/β-catenin signaling pathway and EMT [34]. Consistently, we also found that RIPK4 acted as an oncogene in the progression of OC. Interestingly, increasing evidence indicated that Wnt/β-catenin signaling pathway has been found to play an critical role in many oncogenic processes in OC, including tumorigenesis, metastasis, recurrence, and chemotherapy resistance [35]. NF-κB signaling pathway has also been verified to participate in chemoresistance, cancer stem cell maintenance, metastasis and immune evasion of OC [36]. Considering that these pathways play important roles in OC progression, we speculate that RIPK4 may promote OC progression through these signaling pathways.

## Conclusion

To sum up, our findings indicated that miR-330-3p suppressed the progression of OC by targeting RIPK4. Our results indicated that miR-330-3p/RIPK4 axis participated in the progression of OC, which might provide a novel therapeutic target for OC treatment.

## Highlights

1. miR-330-3p was downregulated in OC tissue specimens and cell lines.

2. Overexpression of miR-330-3p suppressed the proliferation, migration and invasion of OC cells.

3. Overexpression of miR-330-3p suppressed the proliferation, migration and invasion of OC cells via RIPK4.
